# *Helicobacter pylori* Infection Is Associated with Higher CD4 T Cell Counts and Lower HIV-1 Viral Loads in ART-Naïve HIV-Positive Patients in Ghana

**DOI:** 10.1371/journal.pone.0143388

**Published:** 2015-11-24

**Authors:** Fred Stephen Sarfo, Kirsten Alexandra Eberhardt, Albert Dompreh, Edmund Osei Kuffour, Mareike Soltau, Marei Schachscheider, Jan Felix Drexler, Anna Maria Eis-Hübinger, Dieter Häussinger, Emelia Efua Oteng-Seifah, George Bedu-Addo, Richard Odame Phillips, Betty Norman, Gerd Burchard, Torsten Feldt

**Affiliations:** 1 Kwame Nkrumah University of Science and Technology, Kumasi, Ghana; 2 Komfo Anokye Teaching Hospital, Kumasi, Ghana; 3 Clinical Research Unit, Bernhard Nocht Institute for Tropical Medicine, Hamburg, Germany; 4 Clinic of Gastroenterology, Hepatology and Infectious Diseases, University Hospital Düsseldorf, Düsseldorf, Germany; 5 Institute of Virology, University of Bonn Medical Centre, Bonn, Germany; 6 Kumasi Centre for Collaborative Research, Kumasi, Ghana; University of Pittsburgh Center for Vaccine Research, UNITED STATES

## Abstract

**Background:**

Worldwide, there is a high co-endemicity of HIV and *H*. *pylori* infection and there is growing evidence that *H*. *pylori* co-infection is associated with parameters of HIV disease progression. The objective of this study was to investigate the prevalence of *H*. *pylori* infection, and the association with clinical, immunological and virological parameters in a large cohort of HIV-infected individuals and uninfected controls in a West African country.

**Methods:**

HIV-patients (n = 1,095) and HIV-negative individuals (n = 107) were recruited at a university hospital in Ghana. *H*. *pylori* status was determined using stool antigen testing. HIV-related, clinical and socio-demographic parameters were recorded and analyzed according to *H*. *pylori* status.

**Results:**

The prevalence of *H*. *pylori* infection was significantly lower in HIV-positive compared to HIV-negative individuals (51.5 vs. 88%, p<0.0001). In HIV patients, *H*. *pylori* prevalence decreased in parallel with CD4+ T cell counts. In ART-naïve HIV-infected individuals, but not in those taking ART, *H*. *pylori* infection was associated with higher CD4 cell counts (312 vs. 189 cells/μL, p<0.0001) and lower HIV-1 viral loads (4.92 vs. 5.21 log10 copies/mL, p = 0.006). The findings could not be explained by socio-demographic confounders or reported use of antibiotics. Having no access to tap water and higher CD4+ T cell counts were identified as risk factors for *H*. *pylori* infection.

**Conclusions:**

*H*. *pylori* prevalence was inversely correlated with the degree of immunosuppression. In ART-naïve individuals, *H*. *pylori* infection is associated with favorable immunological and virological parameters. The underlying mechanisms for this association are unclear and warrant investigation.

## Introduction

Recently, the interplay between the Human immunodeficiency virus (HIV) and *Helicobacter pylori* (*H*. *pylori*) infection has attracted attention. A number of studies have reported lower *H*. *pylori* prevalence rates in HIV-infected compared to HIV-negative individuals [1,2]. This association is unexpected, since usually chronic infections are more commonly found in patients with advanced HIV disease. Furthermore, *H*. *pylori* infection is considered a disease of poverty, and poor socioeconomic status has been associated with rather disadvantageous outcomes of HIV infection [3]. The underlying mechanisms for this observed association are unclear. Most existing studies have important limitations such as small sample sizes thus preventing subgroup analyses and robust adjustment for confounders. In particular, information on socio-economic variables, as putative confounders for *H*.*pylori* status is sorely lacking. As a consequence, interpretation and comparison of results are difficult and data published to date is partly inconsistent [2].

Considering the significant epidemiological and pathophysiological overlap of HIV and *H*. *pylori* infection, the investigation of possible interplay is of interest. Over the past few years it has become clear, that the gastrointestinal tract (GIT) plays an important role in the pathophysiology of HIV/AIDS. Chronic immune activation, associated with intestinal barrier dysfunction, has been identified as central pathomechanism in HIV disease [4]. *H*. *pylori* colonize the gastric and duodenal mucosae and induce a specific local and also systemic immune response, involving, among others, CD4+ T cells, dendritic cells, regulatory T cells (Treg) and Th17 cells, with all of these also playing a role in HIV pathogenesis [5–8].

The association of HIV and *H*. *pylori* co-infection has not been systematically studied in sub-Saharan Africa, where more than two thirds of HIV-infected individuals live, and where, at the same time, the vast majority of the population gets infected with *H*. *pylori* during childhood [9–11]. The objective of this study was to investigate the prevalence of *H*. *pylori* infection, and its association with clinical, immunological and virological parameters in a large cohort of HIV-infected individuals and uninfected controls in a West African country.

## Materials & Methods

### Study setting and recruitment

This cross-sectional study was conducted at the Komfo Anokye Teaching Hospital, a tertiary referral hospital in the Ashanti Region of Ghana. Between November 2011 and November 2012, consecutive adult HIV-infected patients presenting to the HIV outpatient clinic, and HIV-negative blood donors presenting to the blood bank of the hospital, were offered participation in the study. All participants gave a written informed consent prior to enrolment. The study was conducted in conformity with the Helsinki declaration, and was approved by the appropriate ethics committees of the Kwame Nkrumah University of Science and Technology (Ghana) and of the medical association in Hamburg (Germany).

### Data collection and measures

Demographic, socioeconomic, and clinical data, as well as a detailed medical history were recorded using standardized questionnaires, which were completed by trained study personnel. In particular, time since diagnosis of HIV infection, duration and kind of antiretroviral therapy (ART), co-medications, and clinical parameters were documented. Routine laboratory parameters were extracted from patient’s folders. EDTA blood samples were obtained for the analysis of CD4/CD8 T cell counts, using a FACSCalibur® flow cytometer (Becton Dickinson, USA). HIV-1 and 2 antibody testing was done using the First Response® HIV-1/2 test (Premier Medical Corporation Limited, India) and the Genscreen® ULTRA HIV Ag-Ab Assay (Bio-Rad, France). EDTA plasma and native stool samples were freshly frozen at -80°C and transported to Germany on dry ice. Stool was tested for *H*. *pylori* using the RidaScreen® FemtoLab *H*. *pylori* stool antigen test (R-Biopharm AG, Germany). The sensitivity and specificity of this test has been described to be 98% and 96.7% in pediatric patients and 93% and 90% in adult patients [12,13]. HIV-1 viral load was measured using the RealTime HIV-1 PCR system (Abbott Diagnostics, Wiesbaden, Germany) according to the manufacturer’s instructions. The same tests, except HIV-1 viral load analysis, were conducted for cases and controls.

### Statistical analysis

Parametric variables were compared using the Student’s t-test, non-parametric variables were compared using the Mann-Whitney U-test. Categorical data were analyzed using Chi-squared or Fisher’s exact test. A multivariable logistic regression model was used to analyze the association between *H*. *pylori* infection and other demographic, clinical and laboratory parameters, using only parameters with a significance level of ≤0.05 in bivariate analysis and a correlation coefficient of ≤0.10 in the multivariate regression model. Missing data were excluded from analysis. Statistical analyses were conducted with SPSS version 19 software (IBM, Germany).

## Results

### Cohort characteristics

We recruited 1,095 HIV-positive individuals and 107 HIV-negative blood donors. Stool samples for *H*. *pylori* testing were available for 952 HIV-positive (86.9%) and 100 HIV-negative individuals (93.5%). HIV-positive, compared to HIV-negative individuals, were more often female, significantly older, had a lower BMI, lower socioeconomic status, lower CD4 and higher CD8 T cell counts (Table 1). The majority of HIV-infected participants were female (75.6%), and the mean age was 40 years. Approximately half of HIV-positive individuals (n = 500, 52.5%) were ART-naïve at the time of recruitment, 452 (47.5%) patients were receiving ART for a median duration of 45 months (IQR 19–69). Participants receiving ART, compared to ART-naïve participants, were more likely to be female, had a higher BMI, higher total absolute lymphocyte and CD4 T cell counts compared to ART-naïve HIV-positive participants (S1 Table).

**Table 1:** Comparison of demographic and laboratory characteristics of HIV-positive and HIV-negative participants.

Variable	HIV-positive	HIV-negative	p-value
	N = 952	N = 100	
Female gender, n (%)	720 (75.6)	66 (66.0)	0.04
Age (years), mean ± SD	40 ± 9.5	33 ± 12.3	<0.0001
Religion, n (%)^#^			0.12
Christian	814 (85.5)	86 (92.5)	
Moslem	120 (12.6)	6 (6.5)	
Traditional African religion	2 (0.2)	0 (0.0)	
Other	16 (1.7)	1(1.0)	
Educational level, n (%)^#^			<0.0001
Primary education	156 (16.4)	9 (9.7)	
Junior Secondary School	426 (44.7)	7 (7.5)	
Senior Secondary School	133 (14.0)	56 (60.2)	
Tertiary education	51 (5.4)	14 (15.1)	
No formal education	186 (19.5)	7 (7.5)	
Occupation, n (%)^#^			<0.0001
House wife	13 (1.4)	1(1.1)	
Farmer	78 (8.2)	2 (2.2)	
Trader	505 (53.0)	33 (35.5)	
Salary worker	60 (6.3)	27 (29.0)	
Others	114 (12.0)	4 (4.3)	
Currently unemployed	182 (19.1)	24 (25.8)	
Access to tap water, n (%)*	501 (52.6)	61 (63.5)	0.04
*H*. *pylori* test result, n (%)			
Positive	490 (51.5)	88 (88.0)	<0.0001
Negative	452 (47.5)	12 (12.0)	
Indeterminate	10 (1.0)	0 (0.0)	
BMI (kg/m^2^), mean ± SD	23.1 ± 4.6	24.7 ± 5.0	0.002
T-cell populations, median (IQR)			
Total T-cell count/μL	1,381 (984–1,968)	1,460 (1,171–1,895)	0.13
CD4 T-cell count/μL	380 (173–596)	958 (786–1,161)	<0.0001
CD8 T-cell count/μL	914 (620–1,341)	439 (312–673)	<0.0001

BMI, Body mass index

^#^ missing data for 7 participants of the HIV negative group.

* Missing data for 4 participants of the HIV-negative group.

### *H*. *pylori* infection

**Fig 1:**
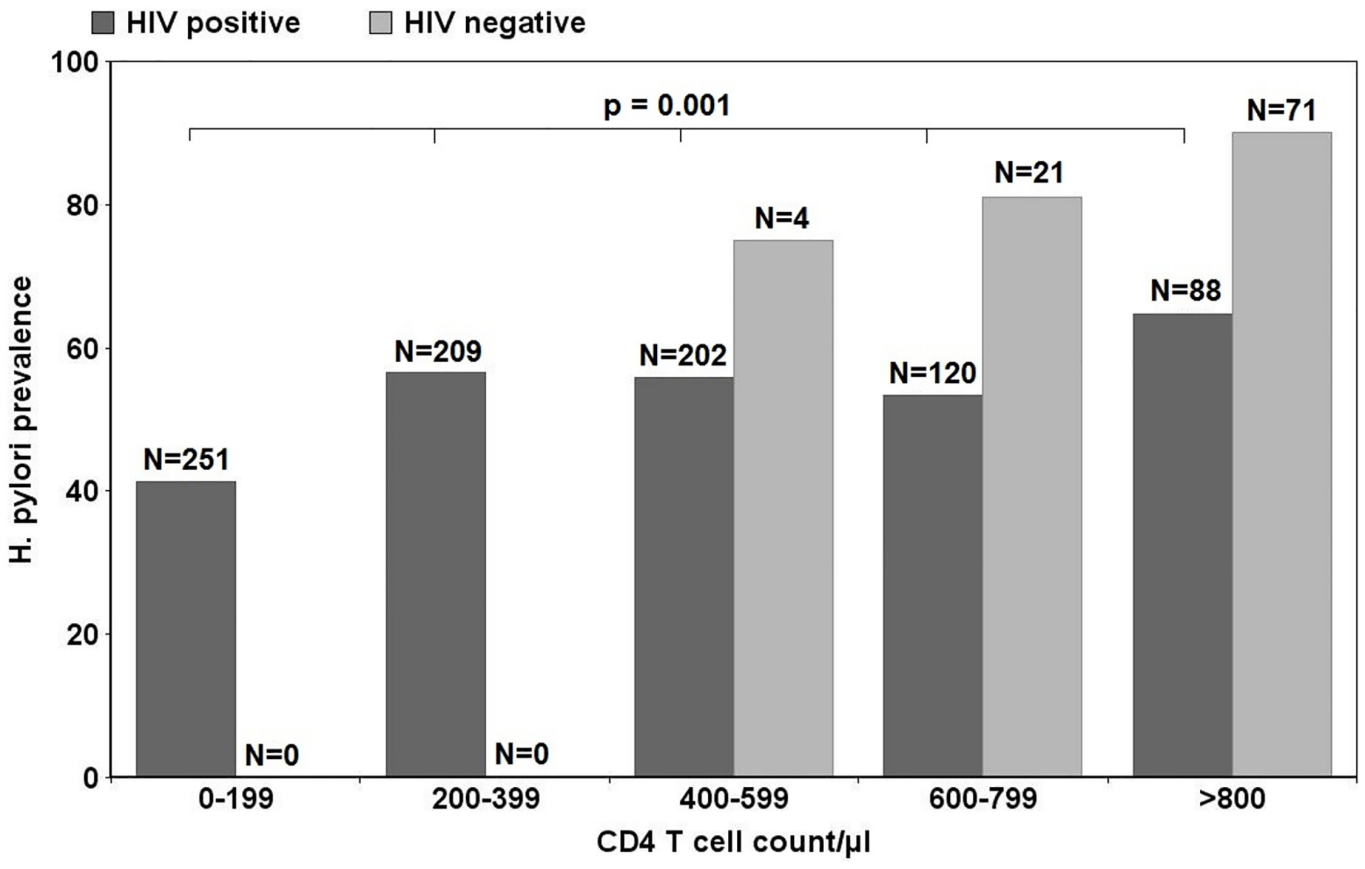
Comparison of *H*. *pylori* prevalence according to CD4 T cell count/μL for HIV-positive participants (p = 0.001, Chi-square test) and for HIV-negative individuals (p = 0.397, Chi-square test); N = Group sizes for CD4 T cell categories including *H*. *pylori* positive and negative participants.

The prevalence of *H*. *pylori* infection among HIV-negative individuals was significantly higher compared to HIV-positive individuals (88.0% vs. 51.5%, p<0.0001). In HIV-positive individuals, *H*. *pylori* prevalence declined in parallel with CD4+ T cell counts, from 64.8% in patients with more than 800 CD4 T cells/μL, to 41.4% in patients with less than 200 CD4 T cells/μL. The same trend was observed in HIV-negative individuals, without reaching statistical significance (Fig 1).

The characteristics of HIV-infected individuals according to *H*. *pylori* status are shown in Table 2. *H*. *pylori* co-infected HIV-positive patients were significantly less likely to have access to tap water (48.8 vs. 58.0%, p = 0.005) and less likely to have attained a tertiary level education (3.7 vs. 7.3%, p = 0.01). There were no significant differences in other demographic variables assessed, or in WHO clinical HIV disease stages (Table 3).

**Table 2:** Comparison of socio-demographic parameters of HIV-infected participants according to *H*. *pylori* status.

Variable	*H*. *pylori* positive	*H*. *pylori* negative	p-value
	N = 490	N = 452	
Female gender, n (%)	372 (75.9)	339 (75.0)	0.74
Age (years), mean ± SD	40 ± 9.4	40 ± 9.6	0.97
Religion, n (%)^#^			
Christian	410 (83.7)	396 (87.6)	0.12
Moslem	71 (14.5)	47 (10.4)	
Traditional African religion	2 (0.4)	0 (0.0)	
Other	7 (1.4)	9 (2.0)	
Educational level, n (%)^#^			0.08
No formal education	91 (18.6)	91 (20.1)	
Primary education	84 (17.1)	72 (15.9)	
Secondary education	297 (60.6)	256 (56.6)	
Tertiary education	18 (3.7)	33 (7.3)	
Occupation, n (%)^##^			0.75
House wife	6 (1.2)	7 (1.5)	
Farmer	42 (8.6)	36 (8.0)	
Trader	257 (52.4)	242 (53.5)	
Salary worker	27 (5.5)	33 (7.3)	
Others	64 (13.1)	48 (10.6)	
Currently unemployed	94 (19.2)	86 (19.0)	
Access to tap water, n (%)	239 (48.8)	262 (58.0)	0.005
Electricity in the household, n (%)	452 (92.2)	420 (92.9)	0.39
Television in household, n (%)	398 (81.2)	366 (81.0)	0.92
Owning a fridge, n (%)	336 (68.6)	322 (71.2)	0.89
Owning a car, n (%)	35 (7.1)	51 (11.3)	0.03

Analysis excludes 10 patients with indeterminate H. pylori result.

**Table 3:** Comparison of clinical and laboratory parameters in HIV-positive and HIV-negative individuals according to H. pylori status.

Variable	ART-naïve group, n = 494			ART group, n = 448			HIV negative group, n = 100		
	*H*. *pylori* pos.	*H*. *pylori* neg.	p-value	*H*. *pylori* pos.	*H*. *pylori* neg.	p-value	*H*. *pylori* pos.	*H*. *pylori* neg.	p-value
	N = 239 (48.4%)	N = 255 (51.6%)		N = 251 (56.0%)	N = 197 (44.0%)		N = 88 (88.0%)	N = 12 (12.0%)	
**Time since HIV diagnosis** (months), median (IQR)	0.5 (0.5–3.5)	0.5 (0.5–1.0)	0.006	53 (24–82)	53 (25–74)	0.42	NA	NA	NA
**Time on ART** (months) median (IQR)	NA	NA		45 (18–70)	47 (22–68)	0.98	NA	NA	NA
**WHO stage** ^#^			0.22			0.15	NA	NA	NA
1	109 (45.6)	118 (46.3)		139 (55.4)	117 (59.4)		NA	NA	
2	23 (9.6)	31 (12.2)		34 (13.5)	13 (6.6)		NA	NA	
3	28 (11.7)	38 (14.9)		35 (13.9)	28 (14.2)		NA	NA	
4	0 (0.0)	2 (0.8)		2 (0.8)	4 (2.0)		NA	NA	
No data	79 (33.1)	66 (25.9)		41 (16.3)	35 (17.8)		NA	NA	
**Exposure to TB treatment**, n (%)	21 (8.8)	41 (16.1)	0.01	24 (9.6)	17 (8.6)	0.87	0 (0.0)	0 (0.0)	NA
Currently on TB treatment, n (%)	8 (3.3)	25 (9.8)	0.004	1 (0.4)	2 (1.0)	0.58	0 (0.0)	0 (0.0)	NA
Previous TB treatment, n (%)	13 (5.4)	16 (6.3)	0.69	23 (9.2)	15 (7.6)	0.56	0 (0.0)	0 (0.0)	NA
**Antibiotic use**									NA
Antibiotic use past six months, n (%)	3 (1.3)	2 (0.8)	0.68	0 (0.0)	1 (O.5)	0.58	0 (0.0)	0 (0.0)	
Current use of co-trimoxazole, n (%)	62 (25.9)	80 (31.4)	0.18	49 (19.5)	48 (24.4)	0.25	0 (0.0)	0 (0.0)	
**Self-reported symptoms** *									
Epigastric discomfort	24 (10.0)	24 (9.4)	0.81	5 (2.0)	8 (4.1)	0.20	18 (21.4)	0 (0.0)	0.08
Anorexia	6 (2.5)	12 (4.7)	0.19	2 (0.8)	5 (2.5)	0.14	0 (0.0)	0 (0.0)	NA
Nausea and vomiting	9 (3.8)	16 (6.3)	0.11	2 (0.8)	6 (3.0)	0.07	2 (2.4)	0 (0.0)	0.68
Diarrhea	23 (9.6)	23 (9.0)	0.82	5 (2.0)	1 (0.5)	0.17	12 (14.3)	2 (16.7)	0.83
Weight loss	78 (32.6)	102 (40.0)	0.09	15 (6.0)	16 (8.1)	0.37	4 (4.5)	0 (0.0)	0.83
**Body Mass Index** (kg/m^2^), mean ±SD	22.4 ± 4.1	21.9 ± 4.2	0.19	24.3 ± 4.5	23.8 ± 4.6	0.21	24.7 ± 5.0	24.6 ± 5.6	0.96
**HIV-1 VL (**log 10 c/mL), median (IQR)^§^	4.92 (4.09–5.51)	5.21 (4.59–5.63)	0.006	3.67 (3.10–4.57)	3.09 (2.31–4.71)	0.54	NA	NA	NA
**T-cell populations**, median (IQR)									
Total T-cell count/μL	1227 (867–1929)	1253(794–1921)	0.94	1452 (1108–1934)	1584 (1151–2083)	0.05	1439 (1166–1910)	1520 (1318–1717)	0.79
CD4 T-cell count/μL	312 (128–508)	189 (75–403)	<0.0001	450 (270–643)	476 (272–654)	0.78	977 (792–1205)	861 (741–1008)	0.23
CD8 T-cell count/μL	832 (564–1336)	980 (595–1569)	0.29	858 (610–1230)	990 (697–1356)	0.02	436 (309–637)	585 (402–758)	0.18
CD4/CD8 ratio	0.31 (0.17–0.60)	0.19 (0.09–0.41)	<0.0001	0.55 (0.37–0.84)	0.49 (0.32–0.81)	0.07	2.12 (1.70–2.91)	1.80 (1.01–2.42)	0.13
**WBC** (x1000/μL), mean ± SD	4.99 ± 1.98	5.28 ± 2.09	0.35	5.0 (4.0–6.0)	5.0 (4.0–6.0)	0.90	NA	NA	NA
**Hemoglobin** (g/dL), mean ± SD	11.1 ± 1.79	10.4 ± 2.02	0.01	12.0 (11.0–13.0)	12.0 (11.0–13.0)	0.83	NA	NA	NA
**Platelets** (x1000/μL), mean ± SD	262.4 ± 97.2	314.9 ± 124.2	0.003	283 (224–333)	283 (228–330)	0.84	NA	NA	NA

Analysis excludes 10 patients with indeterminate *H*. *pylori* result. BMI, Body mass index; WBC, White blood cells; Hgb, Hemoglobin

^#^ WHO clinical stage at recruitment, missing data for 147 patients of the ART-naïve group and 79 of the ART group.

^**§**^ Missing viral load data for 14 H. pylori positive and 17 H. pylori negative participants

*Self-reported symptoms in the past 4 weeks, weight loss defined as significant for the patient, or loss of >10% of body weight. Diarrhea was defined as the passage of three or more loose or liquid stools per day

### Associations between *H*. *pylori* infection and HIV clinical, immunological and virological parameters

Among ART-naïve HIV patients, those with *H*. *pylori* co-infection had higher CD4 T cell counts (312 vs. 189 cells/μl, p<0.0001), higher CD4/CD8 ratios (0.31 vs. 0.19, p<0.0001) and lower HIV-1 viral loads (4.92 vs. 5.21 log10 copies/ml, p = 0.006) compared to those without *H*. *pylori* co-infection. *H*. *pylori* positive patients in this group also had higher mean hemoglobin levels (11.1 vs. 10.4 g/dl, p = 0.01), and lower platelet counts (262.4 vs. 314.9 x1000/μl, p = 0.003), as shown in Table 3. There was no significant difference in the reported use of antibiotics in the 6 months before recruitment between *H*. *pylori* positive and negative individuals.

*H*. *pylori* infection was also not associated to increased frequencies of gastrointestinal symptoms in *H*. *pylori* positive, compared to negative patients, with weight loss (32.6% vs. 40%, p = 0.09), epigastric discomfort (10.0% vs. 9.4%, p = 0.81), and diarrhea (9.6% vs. 9.0%, p = 0.82) being the most common symptoms. In the HIV-infected, ART-exposed group, no significant associations between *H*. *pylori* status and CD4+ T cell count, HIV-1 viral load, or the proportion of patients with undetectable viral load were observed. However, significantly lower CD8+ T cell counts (858/μL vs. 990/μL, p = 0.02), and a trend towards higher CD4/CD8 ratios (0.55 vs. 0.49, p = 0.07), as possible indicator of decreased immune activation, were noted among those patients with *H*. *pylori* co-infection [14–17].

Among HIV-negative controls, no differences in baseline characteristics, symptoms, or socio-demographic parameters were observed between individuals with and without *H*. *pylori* infection. A weak trend towards higher CD4/CD8 ratios was also observed in those HIV-negative individuals with *H*. *pylori* infection, compared to those without *H*. *pylori* infection (2.12 vs. 1.80, p = 0.13).

#### Logistic regression analysis of risk factors associated with H. pylori infection in HIV-positive individuals

Using a logistic multivariable regression model including parameters with p≤0.05 in the univariate analysis and a correlation coefficient of ≤0.1 in the regression model, only CD4+ T cell count (aOR 1.06, 95% CI 1.01–1.12, p = 0.012 for every 100 cells/μl higher) and having access to tap water (aOR 0.63, 95% CI 0.47–0.84, p = 0.002) were associated with *H*. *pylori* infection (Table 4). Significant predictors of *H*. *pylori* co-infection noted in univariate but not in multivariate analysis included use of anti-tuberculous therapy, current use of ART and use of co-trimoxazole. The risk ratio (RR) for *H*. *pylori* infection was 0.82 for those patients having access to tap water and 1.37 for those with >200 CD4 T cells/μl within the group of HIV-positive patients. No risk factors were identified to be associated with *H*. *pylori* infection in the HIV-negative group (data not shown).

**Table 4:** Univariate and multivariate logistic regression analysis of factors associated with *H*. *pylori* co-infection among HIV-infected individuals.

Predictor	Unadjusted OR (95% CI)	p-value	Adjusted OR (95% CI)	p-value
Female gender	0.95 (0.71–1.28)	0.743		
			-	-
Age	0.10 (0.99–1.01)	0.968	-	-
Educational level	0.94 (0.85–1.03)	0.167	-	-
Access to Tap water	0.69 (0.53–0.89)	0.005	0.63 (0.47–0.84)	0.002
Intake of tuberculosis therapy	0.66 (0.43–0.10)	0.049	0.72 (0.46–1.12)	0.142
Use of co-trimoxazole	0.74 (0.55–0.10)	0.046	0.75 (0.53–1.04)	0.084
Use of ART	1.36 (1.05–1.76)	0.019	1.17 (0.86–1.59)	0.331
Duration on ART	1.00 (1.00–1.01)	0.535	-	-
*Each 12-month increase*				
T-cell CD4 count	1.07 (1.03–1.11)	0.001	1.06 (1.01–1.12)	0.012
*Each increase of 100 cells/*μ*L*				
Viral load	0.91 (0.86–0.98)	0.007	-	-
*Each increase of 1 log c/mL*				

Parameters with a p-value ≤0.05 and a correlation coefficient of ≤0.1 between the parameters were included into the multivariate regression model.

## Discussion

This is the first and largest study to systematically investigate the interplay between *H*. *pylori* and HIV infection in sub-Saharan Africa, where both infections are highly co-endemic. We assessed the prevalence of *H*. *pylori* co-infection in a large cohort of unselected adult HIV-infected individuals and HIV-negative controls, and its association with clinical, immunological and virological parameters. We found a graded decrease in *H*. *pylori* prevalence in relation to the level of immune competence, being 88% in HIV-negative and 51.5% in HIV-positive individuals. Among HIV positive individuals, *H*. *pylori* prevalence declined in tandem with CD4+ T cell counts. A similar trend was observed in HIV-negative individuals, although statistical significance was not attained.

Our results are in accordance with previous epidemiologic studies, indicating a lower *H*. *pylori* prevalence in HIV-positive compared to HIV-negative individuals, and also among patients with AIDS compared to matched HIV-infected patients without AIDS [1,2]. However, the interpretation of existing studies is hampered by important limitations, such as small sample sizes which precluded subgroup analyses, and heterogeneous study populations, often including only patients with gastrointestinal symptoms [2]. Information on socio-demographic variables, as putative confounders for *H*. *pylori* status, often lacking in previous studies have been explored in the present study. Furthermore, studies including asymptomatic patients used serological tests to determine *H*. *pylori* status, which have been shown to be problematic especially in HIV-infected individuals [18]. *H*. *pylori* stool antigen tests, as employed in the present study, are non-invasive and have a proven high sensitivity and specificity, making them suitable tools for epidemiologic studies including HIV-infected individuals [12,13].

Although *H*. *pylori* is generally considered a disease of poverty and known to be associated with poor hygienic conditions, HIV-negative participants in our study, having a clearly higher *H*. *pylori* prevalence, ironically had indicators of a higher socioeconomic status. This suggests that the significant differences in *H*. *pylori* prevalence observed between the HIV positive and HIV negative participants may not be explained wholly by socioeconomic disparities. Indeed, the HIV negative participants had more frequent access to tap water compared to HIV-positive individuals, and having no access to tap water was independently associated with the *H*. *pylori* infection in our study. Besides indicating poor sanitary conditions, the lack of access to tap water might also directly promote *H*. *pylori* acquisition by consumption of contaminated drinking water, e.g. from wells. An association between *H*. *pylori* and the consumption of water from wells has previously been reported from India, [19] and *H*. *pylori* has also been identified in drinking water samples from Pakistan by PCR [20].

We also found a significant graded decrease in *H*. *pylori* prevalence with the progression of immunodeficiency in HIV-positive individuals, with the same trend being observed in HIV-negative individuals, but without reaching statistical significance. The underlying mechanisms responsible for this association between immune competence and *H*. *pylori* prevalence are still unclear, although several hypotheses have been offered [2]. The most popular is that more frequent bacterial infections in HIV patients, especially those with advanced disease stages, lead to antibiotic treatment courses, probably resulting in unintended *H*. *pylori* eradication [2]. We found no association between *H*. *pylori* status and reported intake of antibiotics in the past six months before recruitment. Furthermore, only few patients reported taking antibiotics in this period of time, making it unlikely that the observed differences in *H*. *pylori* prevalence are explained by unintended eradication in our study population.

Antibiotic monotherapy has been reported to have only minor efficacy in *H*. *pylori* eradication [21]. Using a meta-analysis methodology, a pooled *H*. *pylori* eradication rate of 19% for monotherapy regimens has been reported [22]. In our study, Co-trimoxazole prophylaxis and tuberculosis therapy were associated with lower risk of *H*. *pylori* status in univariate, but not in multivariate logistic regression analysis. Co-trimoxazole has not been reported to have activity against *H*. *pylori*, and a culture medium containing trimethoprim and sulfamethoxazole has been developed to selectively isolate *H*. *pylori* from animal samples [23]. In contrast, it is known that rifampicin has activity against *H*. *pylori* [24]. A temporary suppression of *H*. *pylori* replication by concurrent tuberculosis treatment, or even clearance of the infection, is thus conceivable. However, it is to be noted that HIV patients with advanced disease are often prescribed Co-trimoxazole prophylaxis against opportunistic infections and are also more likely to receive anti-tubercular therapy for tuberculosis hence the observed lack of significant association in multivariate analyses between use of these antibiotics and risk of *H*. *pylori* co-infection. These findings suggest that progressive HIV disease rather than antibiotic usage may account for the diminution in frequency of *H*. *pylori* co-infection.

Another proposed hypothesis is that the maintenance of *H*. *pylori* infection requires an intact mucosal cellular immunity, and that the loss of the CD4+ T cell population in the gastric mucosa may prevent *H*. *pylori* persistence [2,25,26]. Hence the parallel decline of *H*. *pylori* prevalence with CD4+ T cell count would be consistent with this theory, although there is no evidence that impaired T cell immunity itself might cause a loss of *H*. *pylori* infection. CD4+ T cells have been shown to be increased in *H*. *pylori* gastritis, but gastric inflammation has been shown to correlate with lower *H*. *pylori* bacterial load, and pro-inflammatory genetic profiles are associated to lower *H*. *pylori* seroprevalence [27–29]. While Th1 and Th17-polarized effector T cell subsets are critical for the control of *H*. *pylori* infection, regulatory T cells have the ability to override this T cell driven immunity [30]. Although the alterations of gastric mucosal T cell immunity in the context of HIV infection are incompletely understood, HIV infection apparently rather impairs regulatory T cell suppressive capacity and is thus unlikely to directly promote *H*. *pylori* persistence [31]. Further studies are needed to dissect the interplay between systemic and local mucosal T-cell immunity and *H*. *pylori* persistence in the context of HIV infection.

*H*. *pylori* infection is linked to a number of adverse clinical effects, such as iron deficiency anemia, childhood growth faltering, other gastrointestinal infections and chronic diarrhea[32–35]. In our study however, *H*. *pylori* infection was not associated to the presence of diarrhea, anemia, malnutrition or parasitic diseases (data not shown). Indeed a paradoxical protective effect of *H*. *pylori* infection against tuberculosis has been reported [36]. Furthermore, *H*. *pylori* infection is associated with enhanced Th1-type immune responses to TB antigens [37]. We have recently shown that *H*. *pylori* infection is associated with decreased markers of immune activation in ART-naïve HIV infected patients [38]. Considering that immune activation has been demonstrated to be one of the key mechanisms in HIV pathogenesis [14–17], it is tempting to speculate that *H*. *pylori* infection may influence susceptibility to HIV infection or the natural course of HIV disease. A large proportion of HIV-infected individuals worldwide are co-infected with *H*. *pylori*, hence such interaction could be relevant for the understanding of HIV immunopathology, and could also have public health implications, especially considering the ongoing efforts to develop an *H*. *pylori* vaccine [39].

There are some limitations of our study to be mentioned. The sample size of our HIV-negative group was smaller than that of HIV-positive individuals, and differed in terms of age and gender distribution. However, the main focus of this study was to analyze the effect of *H*. *pylori* within the group of HIV patients. Since we included unselected HIV patients, the group was heterogenous, among others, in terms of ART status and clinical stage of HIV disease. The group of patients taking ART in particular was heterogeneous, and we did not record details on the history and efficacy of ART in terms of CD4+ T cell recovery and virological suppression, limiting the informative value of the analysis in this subgroup. Importantly, the cross sectional study design did not allow for the investigation of causal relationships concerning the described associations.

In conclusion, we have shown that *H*. *pylori* infection is associated with higher CD4+ T cell counts and lower HIV-1 viral loads in ART-naïve patients. Our findings could not be explained by typical confounders as socioeconomic factors, time since diagnosis of HIV infection or unintended *H*. *pylori* eradication by antibiotic use for other infectious conditions. Considering the pathophysiological overlap of both chronic infections, the effects of *H*. *pylori* infection on the systemic immune response, and subsequently on the natural course of HIV disease, warrants further investigation employing prospective studies.

## Supporting Information

S1 Table Comparison of demographic, clinical and laboratory characteristics of HIV positive participants according to ART status. (DOCX)
