# Supplementary material for: Helicobacter pylori Infection Is Associated with Higher CD4 T Cell Counts and Lower HIV-1 Viral Loads in ART-Naïve HIV-Positive Patients in Ghana
Source: PLoS One. 2015 Nov 24;10(11):e0143388. doi: 10.1371/journal.pone.0143388 (PMC4658036; doi:10.1371/journal.pone.0143388)
Supplement: S1 Table — (DOCX) [file pone.0143388.s001.docx]

**S1. Table 1. Comparison of demographic, clinical and laboratory characteristics of HIV positive participants according to ART status**

| **Variable** | **ART- naïve**  **N=500** | **On ART**  **N= 452** | **p** |
| --- | --- | --- | --- |
| Female gender, n (%) | 354 (70.8) | 366 (81.0) | 0.0003 |
| Age (years), mean ± SD | 40 ± 9.7 | 41 ± 9.2 | 0.11 |
| HIV-serotype, n (%)^#^ |  |  | 0.30 |
| HIV-1 | 406 (93.1) | 244 (93.8) |  |
| HIV-2 | 9 (0.9) | 9 (3.5) |  |
| HIV-1 and 2 | 21 (4.8) | 7 (2.7) |  |
| Time since HIV diagnosis (months), median (IQR) | 1 (1 – 3) | 53 (25 – 77) | <0.0001 |
| WHO clinical stage at baseline, n (%)^§^ |  |  | 0.53 |
| 1 | 229 (64.9) | 257 (68.9) |  |
| 2 | 55 (15.6) | 49 (13.1) |  |
| 3 | 67 (19.0) | 63 (17.0) |  |
| 4 | 2 (0.5) | 4 (1.0) |  |
| BMI (kg/m^2^), mean ± SD | 22.2 ± 4.1 | 24.2 ± 5.9 | <0.0001 |
| HIV-1 VL (log10 c/mL), median (IQR) | 5.09 (4.34 – 5.59) | 1.59 (1.59 – 2.01) | <0.0001 |
| Undetectable HIV-1 viraemia^*^, n (%) | 20 (4.4%) | 296 (70.3) | <0.0001 |
| T-cell populations, median (IQR) |  |  |  |
| Total T-cell count/µL | 1,249 (808 – 1,921) | 1,493 (1,109 – 2,008) | <0.0001 |
| CD4 T-cell count/µL | 249 (91 – 450) | 514 (324 – 716) | <0.0001 |
| CD8 T-cell count/µL | 898 (581 – 1,477) | 906 (644 – 1,262) | 0.74 |
| WBC (x1000/µL), median ± SD | 5.2 ± 2.1 | 5.0 ± 1.4 | 0.28 |
| Hgb (g/dL), median ± SD | 10.7 ± 2.0 | 12.0 ± 1.4 | <0.0001 |
| Platelets (x1000/µL), median ± SD | 289.7 ± 116.8 | 282.2 ± 87.4 | 0.41 |
| *H. pylori* test result, n (%) |  |  |  |
| Positive | 239 (47.8) | 251 (55.5) | 0.06 |
| Negative | 255 (51.0) | 197 (43.6) |  |
| Indeterminate | 6 (1.2) | 4 (0.9) |  |
| Antiretroviral therapy (ART), n (%) |  |  |  |
| 2 NRTI + NNRTI | N/A | 437 (96.7) | N/A |
| 2 NRTI + PI | N/A | 15 (3.3) | N/A |
| Time on ART (months), median (IQR) | N/A | 45 (19 – 69) | N/A |

Table 2. BMI, Body mass index; WBC, White blood cells; Hgb, Hemoglobin;

^#^ Missing data for 64 participants of the ART-naïve group and 192 of the ART group.

^§^ WHO clinical stage at recruitment, missing data for 147 patients of the ART-naïve group and 79 of the ART group.

* Undetectable HIV-1 viraemia was defined as viral load <40 copies/mL, corresponding to log 1.59 copies/mL, missing data for 45 participants of the ART-naive group and 31 patients of the ART group.
